# IL-2 in combination with IFN-**α**and 5-FU versus tamoxifen in metastatic renal cell carcinoma: long-term results of a controlled randomized clinical trial

**DOI:** 10.1054/bjoc.2001.2076

**Published:** 2001-10

**Authors:** J Atzpodien, H Kirchner, H J Illiger, B Metzner, D Ukena, H Schott, P J Funke, M Gramatzki, S von Jürgenson, T Wandert, T Patzelt, M Reitz, D. G. C. I. N. (German Cooperative Renal Carcinoma Chemo-Immunotherapy Trials Group)

**Affiliations:** Medizinische Hochschule Hannover, Carl-Neuberg-Str. 1, Hannover, 30625; Europäisches Institut für Tumor-Immunologie und Prävention, Gotenstr. 152, Bonn, 53175; Städtisches Krankenhaus Siloah, Roesebeckstr. 15, Hannover, 30449; Städtische Kliniken Oldenburg, Dr.-Eden-Straβe 10, Oldenburg, 26133; Universitätskliniken des Saarlandes, Oskar-Orth Straβe, Homburg/Saar, 66421; Klinikum Ernst-von-Bergmann, Charlottenstr. 72, Potsdam, 14467; Evangelisches Jung-Stilling-Krankenhaus, Wichernstr. 40, Siegen, 57074; Universitätsklinik Erlangen, Krankenhausstr. 12, Erlangen, 91054, Germany

**Keywords:** renal cancer, interleukin-2, interferon-alpha, tamoxifen

## Abstract

We conducted a prospectively randomized clinical trial to compare the efficacy and safety of subcutaneous interferon-α2a, subcutaneous interleukin-2 and intravenous 5-fluorouracil as home therapy against oral tamoxifen in 78 patients with progressive metastatic renal cell carcinoma. Treatment courses consisted of interferon-α2a 5 × 10^6^ IU m^−2^day 1 weeks 1 + 4; days 1, 3, 5 weeks 2 + 3; 10 × 10^6^ IU m^−2^days 1, 3, 5 weeks 5–8; interleukin-2 10 × 10^6^ IU m^−2^twice daily days 3–5 weeks 1 + 4; 5 × 10^6^ IU m^−2^days 1, 3, 5 weeks 2 + 3; and 5-fluorouracil 1000 mg m^−2^day 1 weeks 5–8. The tamoxifen group received tamoxifen 80 mg twice daily over 8 weeks. Among 41 patients treated with interleukin-2, interferon-α2a and 5-fluorouracil there were 7 complete (17.1%) and 9 partial responders (21.9%), with an overall objective response rate of 39.1% (95% CI, 24.2–55.5). An additional 15 patients (36.6%) were stable throughout therapy. The overall survival was 24 months (range 5–76+). In 37 patients receiving tamoxifen no objective remissions occurred. 13 patients (35.1%) had stable disease and 24 patients (64.9%) showed continued disease progression. The overall survival was 13 months (range 3–73+). In summary, this home-based therapy regimen of interferon-α2a, interleukin-2 and 5-fluorouracil demonstrated significant therapeutic efficacy in patients with progressive renal cell carcinoma when compared to hormonal therapy. © 2001 Cancer Research Campaign  http://www.bjcancer.com


*British Journal of Cancer* (2001) **85**(8), 1130–1136
© 2001 Cancer Research Campaign
doi: 10.1054/ bjoc.2001.2076, available online at http://www.idealibrary.com on IDEAL

http://www.bjcancer.com

### IL-2 in combination with IFN-α and 5-FU versus tamoxifen in metastatic renal cell carcinoma: long-term results of a controlled randomized clinical trial

**J Atzpodien[1,2], H Kirchner[3], HJ Illiger[4], B Metzner[4], D Ukena[5], H Schott[6], PJ Funke[7], M Gramatzki[8], S von Jürgenson[1], T Wandert[2], T Patzelt[2], M Reitz[2] and DGCIN (German Cooperative Renal Carcinoma Chemo-Immunotherapy Trials Group)**

[1]Medizinische Hochschule Hannover, Carl-Neuberg-Str. 1, 30625 Hannover; [2]Europäisches Institut für Tumor-Immunologie und Prävention, Gotenstr. 152, 53175 Bonn; [3]Städtisches Krankenhaus Siloah, Roesebeckstr. 15, 30449 Hannover; [4]Städtische Kliniken Oldenburg, Dr.-Eden-Straβe 10, 26133 Oldenburg; [5]Universitätskliniken des Saarlandes, Oskar-Orth Straβe, 66421 Homburg/Saar; [6]Klinikum Ernst-von-Bergmann, Charlottenstr. 72, 14467 Potsdam; [7]Evangelisches Jung-Stilling-Krankenhaus, Wichernstr. 40, 57074 Siegen; [8]Universitätsklinik Erlangen, Krankenhausstr. 12, 91054 Erlangen, Germany

**Summary** We conducted a prospectively randomized clinical trial to compare the efficacy and safety of subcutaneous interferon-α2a, subcutaneous interleukin-2 and intravenous 5-fluorouracil as home therapy against oral tamoxifen in 78 patients with progressive metastatic renal cell carcinoma. Treatment courses consisted of interferon-α2a $5 \times 10^6$ IU $m^{-2}$, day 1 weeks 1 + 4; days 1, 3, 5 weeks 2 + 3; $10 \times 10^6$ IU $m^{-2}$, days 1, 3, 5 weeks 5–8; interleukin-2 $10 \times 10^6$ IU $m^{-2}$, twice daily days 3–5 weeks 1 + 4; $5 \times 10^6$ IU $m^{-2}$, days 1, 3, 5 weeks 2 + 3; and 5-fluorouracil 1000 mg $m^{-2}$, day 1 weeks 5–8. The tamoxifen group received tamoxifen 80 mg twice daily over 8 weeks. Among 41 patients treated with interleukin-2, interferon-α2a and 5-fluorouracil there were 7 complete (17.1%) and 9 partial responders (21.9%), with an overall objective response rate of 39.1% (95% CI, 24.2–55.5). An additional 15 patients (36.6%) were stable throughout therapy. The overall survival was 24 months (range 5–76+). In 37 patients receiving tamoxifen no objective remissions occurred. 13 patients (35.1%) had stable disease and 24 patients (64.9%) showed continued disease progression. The overall survival was 13 months (range 3–73+). In summary, this home-based therapy regimen of interferon-α2a, interleukin-2 and 5-fluorouracil demonstrated significant therapeutic efficacy in patients with progressive renal cell carcinoma when compared to hormonal therapy. © 2001 Cancer Research Campaign http://www.bjcancer.com

**Keywords**: renal cancer; interleukin-2; interferon-alpha; tamoxifen

Metastatic renal cell carcinoma patients present with a 5-year survival of less than 10%. With little or no efficacy of conventional treatment strategies, including single agent chemotherapy and hormonal therapy, new approaches are needed (Patel and Lavengood, 1978; Krown, 1985; Maldazys and deKernion, 1986). Previously, recombinant cytokines, notably interleukin-2 (IL-2) and interferon-α2a (IFN-α), have shown encouraging results (Rosenberg et al, 1987; West et al, 1987; Figlin et al, 1992). While intravenous bolus or constant infusion recombinant interleukin-2 produces objective responses, this modality is associated with significant potentially life-threatening side effects and considerable expenses (Rosenstein et al, 1986; Lee et al, 1989).

Immunotherapy with subcutaneous recombinant IL-2 alone or in combination with subcutaneous recombinant IFN-α at doses far below the maximum tolerated dose yields significant therapeutic efficacy while reducing treatment-related toxicity (Atzpodien et al, 1990, 1991; Sleijfer et al, 1992). The concomitant use of chemotherapeutic agents, notably 5-fluorouracil (5-FU), further enhances the antineoplastic activity of recombinant cytokines in renal cell carcinoma (Wadler and Schwartz, 1990; Reiter et al, 1992).

*Received 21 February 2001*
*Revised 17 July 2001*
*Accepted 20 July 2001*

*Correspondence to:* J Atzpodien

Tamoxifen is a non-steroidal antioestrogen which is successfully administered in breast cancer patients (Early Breast Cancer Trialists' Collaborative Group, 1992). However, tamoxifen as single-agent appeared to be hardly effective in patients with metastatic renal cell carcinoma (Schomburg et al, 1993).

In this study, we report on a randomized clinical trial of s.c. IFN-α, s.c. IL-2 and i.v. 5-FU compared to single hormonal therapy with tamoxifen in patients with progressive metastatic renal cell cancer.

### PATIENTS AND METHODS

### Patients

Between October 1993 and November 1994, 78 patients (Table 1) with progressive metastatic renal cell carcinoma were entered onto this multicentre randomized clinical trial; median follow up of these patients is 18 months (95% CI 13–23). Pretreatment included radical tumour nephrectomy in all patients ($n$ = 78), palliative surgery ($n$ = 39), radiotherapy ($n$ = 7), hormonal therapy ($n$ = 2), chemotherapy ($n$ = 1) immunotherapy ($n$ = 12), immunochemotherapy ($n$ = 2) and tumourvaccine ($n$ = 11).

Since all treatment regimens were designed to be administrated at home, this required selection of patients with good or excellent

The first author is supported by grants of Deutsche Krebshilfe, Wilhelm-Sander-Stiftung and Gesellschaft zur Förderung immunologischer Krebstherapien e.V.

**Table 1** Patient characteristics and pre-treatment

| Characteristic | All patients | Arm A IL-2/INF-α/5-FU | Arm B tamoxifen |
|---|---|---|---|
| Entered[a] | 78 | 41 | 37 |
| Sex | | | |
| Male | 59 | 25 | 23 |
| Female | 19 | 9 | 9 |
| Age (years) | | | |
| Median | 57 | 54 | 64 |
| Range | 32–78 | 32–72 | 48–78 |
| Pre Treatment | | | |
| Tumornephrectomy | 78 | 41 | 37 |
| Palliative surgery | 39 | 21 | 18 |
| Radiotherapy | 7 | 4 | 3 |
| Hormonal therapy | 2 | 1 | 1 |
| Chemotherapy | 1 | 1 | 0 |
| Immunotherapy | 12 | 5 | 7 |
| sc IL-2 /sc INF-α | 11 | 5 | 6 |
| INF-α | 1 | 0 | 1 |
| Immunochemotherapy | 2 | 2 | 0 |
| sc IL-2 /sc INF-α/iv vinblastine | 1 | 1 | 0 |
| sc INF-α /iv vinblastine | 1 | 1 | 0 |
| Tumorvaccine | 11 | 5 | 6 |
| Others | 2 | 2 | 0 |

[a]Patients entered between October 1993 and November 1994.

**Table 2** Risk stratification models and patient risk distribution[a]

| I Prognostic variables according to Lopez-Hanninen et al (1996) | II Prognostic variables according to Palmer et al (1992) |
|---|---|
| ● Erythrocyte sedimentation rate > 70 mm 1 $h^{-1}$ | ● ECOG performance status > 0 |
| ● Lactic dehydrogenase > 280 U $l^{-1}$ | ● more than one metastatic site |
| ● Neutrophilic granulocytes > 6000 $\mu l^{-1}$ | ● interval from diagnosis of primary tumour to treatment of metastatic disease ≤ 2 years |
| ● Hemoglobin < 10 g $dl^{-1}$ | |
| ● extrapulmonary metastases only | |
| ● bone metastases | |
| Individual risk was defined as | Individual risk was defined as |
| LR low risk in patients without any of these factors | LR low risk in patients with either one or none of these factors |
| HR high risk in patients with one or more of these factors | HR high risk in patients with more than one of these factors |

[a]There was no statistically significant inbalance of risk factors and risk scores when comparing both treatment arms.

performance status. Criteria for entry into the study were: histologically confirmed metastatic renal cell carcinoma; Karnofsky performance status > 80%; white blood cell count > 3500 $\mu l^{-1}$; platelet count > 100 000 $\mu l^{-1}$; haematocrit > 30%; serum creatinine < 1.25 of the upper normal limit; chronic active infection; lack of severe cardiac or pulmonary disease; absence of brain metastases.

The human investigations were performed after approval by a local Human Investigations Committee and in accordance with an assurance filed with and approved by the Department of Health and Human Services; written informed consent was obtained from all patients prior to entry into the study.

### Study design

Patients were stratified according to known clinical predictors (Elson et al, 1988; Tourani et al, 1998) to allow for equal risk distribution in both treatment arms (Table 2, Figure 1). They were subsequently randomized to receive IL-2 and IFN-α combined with 5-FU (Arm A) or tamoxifen (Arm B); randomisation was performed on a per centre basis to rule out centre-related statistical bias. There was no statistically significant inbalance of risk factors and risk scores when comparing both treatment arms.

Patients were treated in an outpatient setting. The immunochemotherapy (Arm A) consist of IFN-α, IL-2 and 5-FU. Sc IFN-α was administrated at $5 \times 10^6$ IU $m^{-2}$, day 1 week 1 + 4, days 1, 3, 5 weeks 2 + 3; and $10 \times 10^6$ IU $m^{-2}$, days 1, 3, 5 weeks 5–8. Sc IL-2 was administrated $10 \times 10^6$ IU $m^{-2}$, twice daily days 3–5 weeks 1 + 4; and $5 \times 10^6$ IU $m^{-2}$, days 1, 3, 5 weeks 2 + 3; additionally, patients received iv 5-FU at 1000 mg $m^{-2}$, day 1 weeks 5–8. The hormonal therapy (Arm B) consisted of 80 mg tamoxifen twice daily. 8-week treatment cycles were repeated for up to 3 courses unless progression of disease occurred.

In the immunochemotherapy group (Arm A) 34% of patients received one cycle, 43% of patients obtained 2 cycles and 23% received 3 cycles. In the hormonal therapy group (Arm B) 72% of patients passed through one cycle, 19% obtained 2 cycles, 6% received 3 cycles, and 3% obtained 4 cycles. Re-evaluation of the patients tumour status was performed between treatment

*© 2001 Cancer Research Campaign*

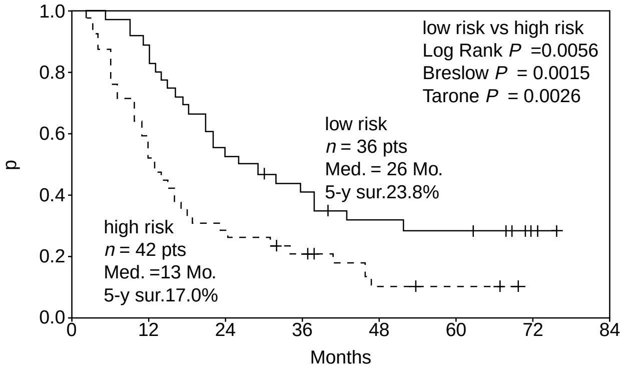

No. of patients at risk

| | | | | | | | |
|---|---|---|---|---|---|---|---|
| LR | 36 | 29 | 19 | 15 | 12 | 11 | 11 |
| HR | 42 | 22 | 10 | 8 | 6 | 6 | – |

**Figure 1** Overall survival (Kaplan–Meier estimates) of 78 patients stratified for risk (Palmer et al, 1992). Survival was measured from start of therapy and tick marks represent censored patients cycles. All patients were examined by means of computed tomography.

After the first 8 weeks of therapy patients primarily treated with p.o. tamoxifen crossed over to immunochemotherapy upon disease progression wherever performance status allowed further systemic therapy. Concomitant medication was given as needed to control adverse effects of immunochemotherapy.

### Assessment of response, survival and toxicity

Response to therapy was evaluated according to World Health Organization (WHO) criteria: complete response – disappearance of all signs of disease for a minimum of 8 weeks; partial response – 50% or more reduction in the sum of products of the greatest perpendicular diameters of measurable lesions, no increase in lesion size and no new lesions; stable disease – less than a partial response with no disease progression for at least 8 weeks; progressive disease – 25% or more increase in sum of products in the longest perpendicular diameters of measurable lesions or the development of new lesions. Duration of response was measured from start of therapy. In case of patients with early progression, progression-free survival was calculated at 0 months. Survival was measured from start of therapy to date of death or to the last known date to be alive.

Systemic toxicity was evaluated at weekly intervals using a grading system adapted from the WHO.

Treatment efficacy was assessed on intent-to-treat basis.

### Statistical analysis

The statistical end points in our analysis were (1) objective response and (2) overall and progression-free survival of patients. The potential response rate was assumed to be 35% (immunochemotherapy) and 5% (hormonal therapy), respectively. It was intended to find an immunochemotherapy-induced objective response benefit over tamoxifen at 30% with a probability of 95% ($\alpha = 0.05$) and a sample power of $1\text{-}\beta = 0.80$. The probability of survival was plotted over time according to the method of Kaplan and Meier. Statistical significance was assessed using the Log-rank, Mantel–Cox and the Breslow test. The Wilcoxon test was used to analyse differences between dependent nonparametric data.

### RESULTS

To balance risk factors by treatment arms patients were stratified according to risk and, subsequently, randomised within risk adjusted groups. 78 patients were enrolled in this clinical trial and stratified for risk. 41 patients were randomized to receive combined immunochemotherapy and 37 patients were randomized to receive single-agent p.o. tamoxifen.

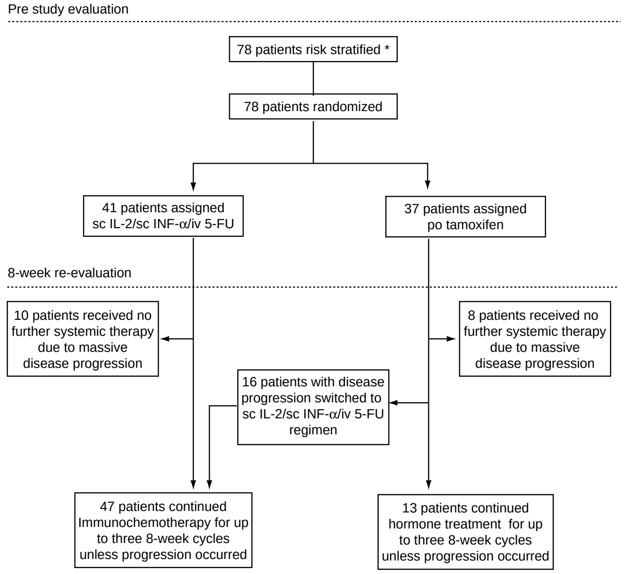

* Initial risk stratification was performed to allow for equal risk distribution in both treatment arms

**Figure 2** Trial profile

 *© 2001 Cancer Research Campaign*

**Table 3** Response to home therapy according to WHO criteria (intent-to-treat)

| **Tumour Site**[b] | **Response** | | | | **Total** |
|---|---|---|---|---|---|
| | **Complete response** | **Partial response** | **Stable disease** | **Progressive disease** | |
| **sc IL-2 /sc INF-α /iv 5-FU (41 patients**[a]**)** | | | | | |
| Lung | 5 | 9 | 14 | 6 | 34 |
| Lymph nodes | 2 | 2 | 7 | 4 | 15 |
| Liver | – | 1 | 3 | 3 | 7 |
| Local relapse | – | – | 2 | 3 | 5 |
| Bone | – | 1 | 3 | 2 | 6 |
| Adrenal gland | – | 1 | – | 1 | 2 |
| Other | 1[c] | – | 4[d] | 3[e] | 8 |
| Total evaluable | 7 | 9 | 15 | 10 | 41 |
| (low/high risk) | (5/2) | (3/2) | (10/4) | (1/9) | (21/20) |
| Percent | 17.1% | 21.9% | 36.6% | 24.4% | 100% |
| | 95%, 24.2%–55.5% | | | | |
| ***po Tamoxifen (37 patients*)*** | | | | | |
| Lung | – | – | 16 | 14 | 30 |
| Lymph nodes | – | – | 7 | 1 | 18 |
| Liver | – | – | 1 | 6 | 7 |
| Local relapse | – | – | 1 | 6 | 7 |
| Bone | – | – | 1 | 8 | 9 |
| Adrenal gland | – | – | – | – | – |
| Other | – | – | 3[f] | 2[g] | 5 |
| Total evaluable | – | – | 13 | 24 | 37 |
| (low/high risk) | (–) | (–) | (9/4) | (6/18) | (15/22) |
| Percent | – | – | 35.1% | 64.9% | 100% |

[a]Patients entered between October 1993 and November 1994; Response was stratified according to Palmer et al (1992).
[b]Patients may have had more than 1 site.
[c]Peritoneum.
[d]Contralateral kidney.
[e]Pleura (*n* = 2); Thoracic wall (*n* = 1).
[f]Contralateral kidney; pleura; Thoracic wall.
[g]Pleura; CNS.

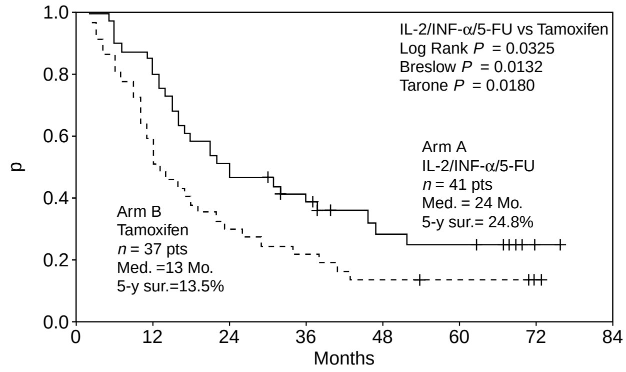

No. of patients at risk

| | | | | | | | |
|---|---|---|---|---|---|---|---|
| Arm A | 41 | 33 | 19 | 16 | 13 | 12 | 12 |
| Arm B | 37 | 19 | 11 | 8 | 5 | 5 | 5 |

**Figure 3** Overall survival (Kaplan–Meier estimates) of 78 patients treated with s.c. interleukin-2, s.c. interferon-α and i.v. 5-fluorouracil or with p.o. tamoxifen. Survival was measured from start of therapy and tick marks represent censored patients

### Treatment response

7 patients (17.1%) treated with s.c. IFN-α, s.c. IL-2 and i.v. 5-FU achieved a complete response and 9 patients (21.9%) had a partial remission (Table 3). The overall objective response rate was 39.1% (95% CI, 24.2–55.5). In partial responders, the median response duration was 18 months (range 3–31), the complete response durations ranged from 3 to 76+ months (median not reached). Objective tumour regressions were seen in lung (14), lymph nodes (4), liver (1), bone (1), adrenal gland (1) and peritoneum (1). 15 patients (36.6 %) showed disease stabilization. The progression-free survival in this subgroup ranged from 1 to 20 months with a median of 7 months. 10 patients (24.4%) exhibited continuous disease progression despite therapy.

In the tamoxifen group, there was no objective response. 13 patients (35.1%) had stable disease with a median progression-free interval of 6 months (range 2–28). 24 patients (64.9%) showed continued progression of disease.

### Overall survival

Overall median survival of all patients entered into the study was 18 months, whereby 17 patients continue to be alive at last follow up. In the immunochemotherapy regimen the median survival was 24 months (range 5–76+; 95% CI 11–37), with statistical significance (Log rank $P = 0.0325$) compared to the tamoxifen group with a median survival of 13 months (range 2–73+; 95% CI 8–18) (Figure 3).

In low-risk patients, there was no statistically significant difference in median survival between the immunochemotherapy group (median survival 32 months; range 5–76+; 95% CI 12–52) and the hormonal therapy group (median survival 26 months; range 9–73+; 95% CI 12–40) (Figure 4). In contrast, high-risk patients yielded a significantly prolonged median survival of 16 months

*© 2001 Cancer Research Campaign* 

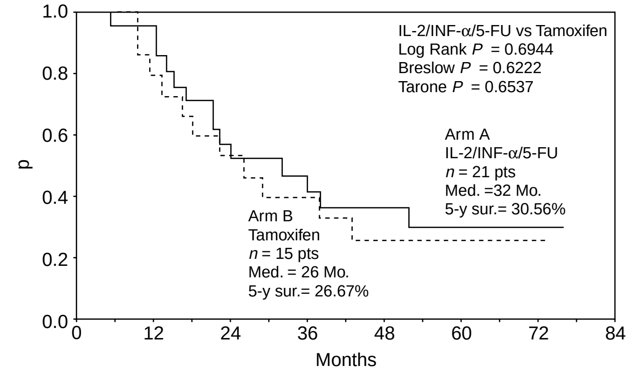

No. of patients at risk

| | | | | | | | |
|---|---|---|---|---|---|---|---|
| Arm A | 21 | 20 | 12 | 9 | 6 | 5 | 2 |
| Arm B | 15 | 12 | 8 | 6 | 4 | 4 | 3 |

**Figure 4** Overall survival (Kaplan–Meier estimates) of 36 low risk patients treated with s.c. interleukin-2, s.c. interferon-α and i.v. 5-fluorouracil or with p.o. tamoxifen. Survival was measured from start of therapy and tick marks represent censored patients

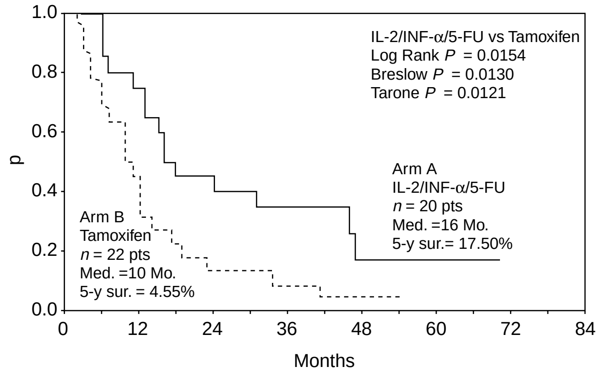

No. of patients at risk

| | | | | | | | |
|---|---|---|---|---|---|---|---|
| Arm A | 20 | 15 | 9 | 6 | 2 | 2 | – |
| Arm B | 22 | 10 | 3 | 2 | 1 | – | – |

**Figure 5** Overall survival (Kaplan–Meier estimates) of 42 high risk patients treated with s.c. interleukin-2, s.c. interferon-α and i.v. 5-fluorouracil or with p.o. tamoxifen. Survival was measured from start of therapy and tick marks represent censored patients

(range 6–70+; 95% CI 12–20) after immunochemotherapy compared to a median survival of 10 months (range 2–54+; 95% CI 7–13) after tamoxifen therapy (Figure 5).

Of 37 patients primarily treated with tamoxifen, 16 with continued progressive disease crossed over to immunochemotherapy regimen and showed an improved median survival of 19 months (range 4–69+; 95% CI 9–29) as opposed to a median survival of 10 months (range 2–72+; 95% CI 8–12) in patients without crossover to secondary immunochemotherapy (Log rank $P = 0.0596$). When compared to the combined immunochemotherapy group, patients primarily treated with tamoxifen showed no statistically significant difference in overall survival after crossing over (Log rank $P = 0.5568$) (Figure 6).

Patients had a 5-year survival of 24.8% with combined immunochemotherapy compared to 13.5% 5-year survival for single-agent tamoxifen (Figure 3). In low-risk patients, there was no difference in 5-year survival between the immunochemotherapy (30.56%) and the tamoxifen group (26.67%); while high-risk patients reached a 5-year survival of 17.50% (immunochemotherapy) and 4.55% (tamoxifen therapy) (Figures 4 and 5).

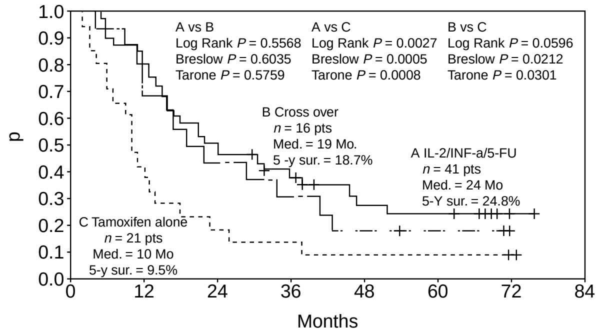

No. of patients at risk

| | | | | | | | |
|---|---|---|---|---|---|---|---|
| A | 41 | 33 | 19 | 16 | 13 | 12 | 12 |
| B | 16 | 10 | 7 | 5 | 3 | 3 | 3 |
| C | 21 | 8 | 6 | 5 | 4 | 4 | 4 |

**Figure 6** Overall survival (Kaplan–Meier estimates) of patients treated with s.c. interleukin-2, s.c. interferon-α and i.v. 5-fluorouracil (41), single agent p.o. tamoxifen (21) and of patients primarily treated with p.o. tamoxifen crossed over to s.c. interleukin-2, s.c. interferon-α and i.v. 5-fluorouracil (16). Survival was measured from start of therapy and tick marks represent censored patients

Patients primarily treated with tamoxifen and crossed over to immunochemotherapy had a 5-year survival of 18.7% as opposed to 9.5% 5-year survival in patients without crossover (Figure 6).

The progression-free survival with a median of 7 months (range 0–76+; 95% CI 3–11) for all patients treated with the combination therapy regimen was significantly prolonged ($P < 0.0001$) compared to the tamoxifen-treated patient group (median 0 months, range 0–28) (Figure 7).

### Treatment toxicity

Combined immunochemotherapy was well tolerated and was always given in the outpatient setting. Side effects were mostly limited to WHO grade I and II with patients experiencing flu-like symptoms as malaise (grade I/II 78%; III 7%), chills (grade I/II

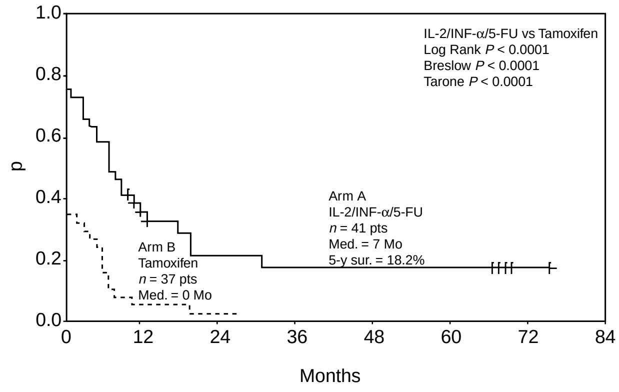

No. of patients at risk

| | | | | | | | |
|---|---|---|---|---|---|---|---|
| Arm A | 41 | 15 | 11 | 10 | 10 | 10 | 10 |
| Arm B | 37 | 2 | 1 | 0 | 0 | 0 | 0 |

**Figure 7** Progression-free survival for all 78 patients entered and treated with s.c. interleukin-2, s.c. interferon-α and i.v. 5-fluorouracil or with single agent p.o. tamoxifen. Plots were generated by the Kaplan–Meier method and tick marks represent censored patients

*© 2001 Cancer Research Campaign*

57%; III 3%), fever (grade I/II 72%; III 2%), nausea/emesis (grade I/II 77%), diarrhoea (grade I/II 34%; grade III 1%) dyspnoea (grade I/II 26%; III 1%), hypotension (grade I/II 23%), mild alopezia (grade I/II 14%) and paresthesias (grade I 9%). In all except 3 patients, therapy was completed without modification of dosage or change of time interval. No toxic deaths occurred. In the tamoxifen group, WHO grade I nausea/emesis (17%) and mild grade I thrombopenia (20%) were observed; no severe tamoxifen-related side effects including hot flushes, deep vein thrombosis or pulmonary embolism did occur (data not shown).

### DISCUSSION

The use of biologic therapies has an established role in the treatment of metastatic renal cell carcinoma (Atzpodien et al, 1990; Wirth, 1991; Figlin et al, 1992; Stahl et al, 1992). However, very few randomized controlled trials have confirmed the therapeutic efficacy of rIL-2 based regimens in this disease.

In the present prospectively randomized trial, we reported the results of 78 patients with progressive metastatic renal cell carcinoma who received (A) combined s.c. rIFN-α, s.c. rIL-2, and i.v. 5-FU or (B) p.o. tamoxifen. Tumour regressions occurred in 39% of patients treated with the combined therapy (17% CRs and 22% PRs). These controlled results were comparable to those of other rIL-2-based therapy regimens in renal cell carcinoma (Sella et al, 1994; Joffe et al, 1996; Tourani et al, 1998). However, in previous phase I/II studies employing our immunochemotherapy regimen response rates of 39% (120 pts, Lopez-Hanninen et al, 1996), 38% (25 pts, Hofmockel et al, 1996), 16% (55 pts, Joffe et al, 1996; Dutcher et al, 1997) and 12% (52 pts, van Herpen et al, 2000), respectively, were reported by 5 different groups. In modified regimens, the combination of IL-2, IFN-α and 5-FU yielded response rates of 31% (Ellerhorst et al, 1997), 20% (Tourani et al, 1998) and 8% (Negrier et al, 1998), respectively, with overlapping 95% confidence intervals from 4% to 56%.

The discrepancy in phase I/II results might be best explained by at least 3 uncontrolled variables: (A) patient selection and clinical/biological risk distribution; (B) variations in the rIL-2 based regimen (e.g., reconstitution of drug, route and dose of administration, use of rIL-2 with concomitant chemotherapy); and (C) different standards of patient care (e.g., surgery, supportive care upon IL-2 therapy).

After tamoxifen therapy some authors reported a low objective response rate of 1.7% (95% CI, 0.04–9.09%) (Schomburg et al, 1993) based on spontaneous regression of metastatic renal cell cancer. The lack of objective remissions in the present tamoxifen group was most likely due to the small number of patients treated.

Previous uncontrolled trials mostly focused on objective responses with limited follow-ups and survival of around 2 years. In the present study we, for the first time, reported the long-term randomized outcome of IL-2-based immunochemotherapy in a risk adjusted cohort of renal cell carcinoma patients.

The median overall survival of 24 months in the immunochemotherapy group was statistically superior to 13 months in the tamoxifen group. Notably, patients failing primary tamoxifen could reach a median survival of 19 months after crossing over to combined immunochemotherapy, and experienced no significant decrease in long-term survival when compared to the primary immunochemotherapy group. This may be due to the fact that only patients with good or fair performance status – and hence possibly slower rate of progression – were crossed over whereas those with lower performance status – and hence more aggressive disease – did not undergo secondary immunotherapy.

Interestingly, survival graphs of low- and high-risk patients, respectively, suggested that high-risk patients experience a more pronounced survival benefit from immunochemotherapy compared to oral tamoxifen.

In most patients reported here, immunochemotherapy-related toxicity was limited to WHO grades I and II, notably malaise, fever, nausea, diarrhoea, while dose-limiting WHO III/IV side effects occurred in no more than 10% of patients and always normalized after termination of therapy. Overall, the s.c. route of application reduced IL-2 toxicity when compared to i.v.-based IL-2 regimens (Sleijfer et al, 1992); also, low therapy-related toxicity as observed here may have been due to adequate patient selection as required for home therapy. In summary, this prospectively randomized clinical trial established the long-term therapeutic efficacy, safety and tolerability of s.c. IL-2, s.c. INF-α, i.v. 5-FU as a home therapy in prospectively controlled patients with metastatic renal cell carcinoma.

### REFERENCES

Atzpodien J, Korfer A, Franks CR, Knuver-Hopf J, Lopez-Hanninen E, Fischer M, Mohr H, Dallmann I and Hadam M (1990) Home therapy with recombinant interleukin-2 and interferon-alpha 2b in advanced human malignancies. *Lancet* **335**: 1509–1512

Atzpodien J, Poliwoda H and Kirchner H (1991) alpha-Interferon and interleukin-2 in renal cell carcinoma: studies in nonhospitalized patients. *Semin Oncol* **18**: 108–112

Atzpodien J, Lopez HE, Kirchner H, Bodenstein H, Pfreundschuh M, Rebmann U, Metzner B, Illiger HJ, Jakse G and Niesel T (1995) Multiinstitutional home-therapy trial of recombinant human interleukin-2 and interferon alfa-2 in progressive metastatic renal cell carcinoma. *J Clin Oncol* **13**: 497–501

Dutcher JP, Atkins M, Fisher R, Weiss G, Margolin K, Aronson F, Sosman J, Lotze M, Gordon M, Logan T and Mier J (1997) Interleukin-2-based therapy for metastatic renal cell cancer: the Cytokine Working Group experience, 1989–1997. *Cancer J Sci Am* **3**(suppl 1): S73–S78

Early Breast Cancer Trialists' Collaborative Group (1992) Systemic treatment of early breast cancer by hormonal, cytotoxic, or immune therapy. 133 randomized trials involving 31,000 recurrences and 24,000 deaths among 75,000 women. *Lancet* **339**: 1–15

Ellerhorst JA, Sella A, Amato RJ, Tu SM, Millikan RE, Finn LD, Banks M and Logothetis CJ (1997) Phase II trial of 5-fluorouracil, interferon-alpha and continuous infusion interleukin-2 for patients with metastatic renal cell carcinoma. *Cancer* **80**: 2128–2132

Elson PJ, Witte RS and Trump DL (1988) Prognostic factors for survival in patients with recurrent or metastatic renal cell carcinoma. *Cancer Res* **48**: 7310–7313

Figlin RA, Belldegrun A, Moldawer N, Zeffren J and deKernion J (1992) Concomitant administration of recombinant human interleukin-2 and recombinant interferon alfa-2A: an active outpatient regimen in metastatic renal cell carcinoma [see comments]. *J Clin Oncol* **10**: 414–421

Hofmockel G, Langer W, Theiss M, Gruss A, and Frohmuller HG (1996) chemo-immunotherapy for metastatic renal cell carcinoma using a regimen of interleukin-2, interferon-alpha and 5-fluorouracil [see comments]. *J Urol* **156**: 18–21

Joffe JK, Banks RE, Forbes MA, Gruss A and Frohmuller HG (1996) A phase II study of interferon-alpha, interleukin-2 and 5-fluorouracil in advanced renal carcinoma: clinical data and laboratory evidence of protease activation. *Br J Urol* **77**: 638–649

Krown SE (1985) Therapeutic options in renal-cell carcinoma. *Semin Oncol* **12**: 13–17

Lee RE, Lotze MT, Skibber JM, Tucker E, Bonow RO, Ognibene FP, Carrasquillo JA, Shelhamer JH, Parrillo JE and Rosenberg SA (1989) Cardiorespiratory effects of immunotherapy with interleukin-2. *J Clin Oncol* **7**: 7–20

Lopez-Hanninen E, Kirchner H and Atzpodien J (1996) Interleukin-2 based home therapy of metastatic renal cell carcinoma: risks and benefits in 215 consecutive single institution patients. *J Urol* **155**: 19–25

*© 2001 Cancer Research Campaign* 

Maldazys JD and deKernion JB (1986) Prognostic factors in metastatic renal carcinoma. *J Urol* **136**: 376–379
Negrier S, Escudier B, Lasset C, Douillard JY, Savary J, Chevreau C, Ravaud A, Mercatelllo A, Peny J, Mousseau M, Philip T and Tursz T (1998) Recombinant human interleukin-2, recombinant human interferon alfa-2a, or both in metastatic renal-cell carcinoma. Groupe Francais d'Immunotherapie [see comments]. *N Engl J Med* **338**: 1272–1278
Patel NP and Lavengood RW (1978) Renal cell carcinoma: natural history and results of treatment. *J Urol* **119**: 722–726
Reiter Z, Ozes ON, Blatt LM and Taylor MW (1992) A dual anti-tumor effect of a combination of interferon-alpha or interleukin-2 and 5-fluorouracil on natural killer (NK) cell-mediated cytotoxicity. *Clin Immunol Immunopathol* **62**: 103–111
Rosenberg SA, Lotze MT, Muul LM, Chang AE, Avis FP, Leitman S, Marston Linehan W, Robertson CN, Lee RE, Rubin JT, Seipp CA, Simpson CG and White DE (1987) A progress report on the treatment of 157 patients with advanced cancer using lymphokine-activated killer cells and interleukin-2 or high-dose interleukin-2 alone. *N Engl J Med* **316**: 889–897
Rosenstein M, Ettinghausen SE and Rosenberg SA (1986) Extravasation of intravascular fluid mediated by the systemic administration of recombinant interleukin 2. *J Immunol* **137**: 1735–1742
Schomburg A, Kirchner H, Fenner M, Menzel T, Poliwoda H and Atzpodien J (1993) Lack of therapeutic efficacy of tamoxifen in advanced renal cell carcinoma. *Eur J Cancer* **29A**: 737–740
Sella A, Kilbourn RG, Gray I, Finn L, Zukiwski AA, Ellerhorst J, Amato RJ and Logothetis CJ (1994) Phase I study of interleukin-2 combined with interferon-alpha and 5-fluorouracil in patients with metastatic renal cell cancer. *Cancer Biother* **9**: 103–111
Sleijfer DT, Janssen RA, Buter, de Vries EG, Willemse PH and Mulder NH (1992) Phase II study of subcutaneous interleukin-2 in unselected patients with advanced renal cell cancer on an outpatient basis. *J Clin Oncol* **10**: 1119–1123
Stahl M, Wilke HJ, Seeber S and Schmoll HJ (1992) Cytokines and cytotoxic agents in renal cell carcinoma: a review. *Semin Oncol* **19**: 70–79
Tourani JM, Pfister C, Berdah JF, Benhammouda A, Salze P, Monnier A, Paule B, Guillet P, Chretien Y, Brewer Y, Di Palma M, Untereiner M, Malaurie E, Tadrist Z, Pavolitch JM, Hauteville D, Mejean A, Azagury M, Mayeur D, Lucas V, Krakowski I, Larregain-Fournier D, Abourachid H, Andrieu JMN and Chastang C (1998) Outpatient treatment with subcutaneous interleukin-2 and interferon alfa administration in combination with flurouracil in patients with metastatic renal cell carcinoma: results of a sequential nonrandomized phase II study. Subcutaneous Administration Propeukin Program Cooperative Group. *J Clin Oncol* **16**: 2505–2513
van Herpen CM, Jansen RL, Kruit WH, Hoekman K, Groenewegen G, Osanto S and de Mulder PH (2000) Chemoimmunotherapy with interleukin-2, interferon-alpha and 5-fluorouracil for progressive metastatic renal cell carcinoma: a multicenter phase II study. Dutch Immunotherapy Working Party. *Br J Cancer* **82**: 772–776
Wadler S and Schwartz EL (1990) Antineoplastic activity of the combination of interferon and cytotoxic agents against experimental and human malignancies: a review. *Cancer Res* **50**: 3473–3486
West WH, Tauer KW, Yannelli JR, Marshall GD, Orr DW, Thurman GB and Oldham RK (1987) Constant-infusion recombinant interleukin-2 in adoptive immunotherapy of advanced cancer. *N Engl J Med* **316**: 898–905
Wirth M (1991) The current use of interferons, interleukin-2 and tumor necrosis factor in renal cell cancer. *Urol Int* **47**: 219–230

 *© 2001 Cancer Research Campaign*

## References

[PDF_00670] Atzpodien J., Körfer A., Franks C. R., Poliwoda H., Kirchner H. (1990). Home therapy with recombinant interleukin-2 and interferon-alpha 2b in advanced human malignancies.. Lancet.

[PDF_00677] Atzpodien J., Lopez Hänninen E., Kirchner H., Bodenstein H., Pfreundschuh M., Rebmann U., Metzner B., Illiger H. J., Jakse G., Niesel T. (1995). Multiinstitutional home-therapy trial of recombinant human interleukin-2 and interferon alfa-2 in progressive metastatic renal cell carcinoma.. J Clin Oncol.

[PDF_00674] Atzpodien J., Poliwoda H., Kirchner H. (1991). alpha-Interferon and interleukin-2 in renal cell carcinoma: studies in nonhospitalized patients.. Semin Oncol.

[PDF_00681] Dutcher J. P., Atkins M., Fisher R., Weiss G., Margolin K., Aronson F., Sosman J., Lotze M., Gordon M., Logan T. (1997). Interleukin-2-based therapy for metastatic renal cell cancer: the Cytokine Working Group experience, 1989-1997.. Cancer J Sci Am.

[PDF_00689] Ellerhorst J. A., Sella A., Amato R. J., Tu S. M., Millikan R. E., Finn L. D., Banks M., Logothetis C. J. (1997). Phase II trial of 5-fluorouracil, interferon-alpha and continuous infusion interleukin-2 for patients with metastatic renal cell carcinoma.. Cancer.

[PDF_00693] Elson P. J., Witte R. S., Trump D. L. (1988). Prognostic factors for survival in patients with recurrent or metastatic renal cell carcinoma.. Cancer Res.

[PDF_00695] Figlin R. A., Belldegrun A., Moldawer N., Zeffren J., deKernion J. (1992). Concomitant administration of recombinant human interleukin-2 and recombinant interferon alfa-2A: an active outpatient regimen in metastatic renal cell carcinoma.. J Clin Oncol.

[PDF_00699] Hofmockel G., Langer W., Theiss M., Gruss A., Frohmüller H. G. (1996). Immunochemotherapy for metastatic renal cell carcinoma using a regimen of interleukin-2, interferon-alpha and 5-fluorouracil.. J Urol.

[PDF_00703] Joffe J. K., Banks R. E., Forbes M. A., Hallam S., Jenkins A., Patel P. M., Hall G. D., Velikova G., Adams J., Crossley A. (1996). A phase II study of interferon-alpha, interleukin-2 and 5-fluorouracil in advanced renal carcinoma: clinical data and laboratory evidence of protease activation.. Br J Urol.

[PDF_00707] Krown S. E. (1985). Therapeutic options in renal-cell carcinoma.. Semin Oncol.

[PDF_00709] Lee R. E., Lotze M. T., Skibber J. M., Tucker E., Bonow R. O., Ognibene F. P., Carrasquillo J. A., Shelhamer J. H., Parrillo J. E., Rosenberg S. A. (1989). Cardiorespiratory effects of immunotherapy with interleukin-2.. J Clin Oncol.

[PDF_00712] Lopez Hänninen E., Kirchner H., Atzpodien J. (1996). Interleukin-2 based home therapy of metastatic renal cell carcinoma: risks and benefits in 215 consecutive single institution patients.. J Urol.

[PDF_00717] Maldazys J. D., deKernion J. B. (1986). Prognostic factors in metastatic renal carcinoma.. J Urol.

[PDF_00719] Negrier S., Escudier B., Lasset C., Douillard J. Y., Savary J., Chevreau C., Ravaud A., Mercatello A., Peny J., Mousseau M. (1998). Recombinant human interleukin-2, recombinant human interferon alfa-2a, or both in metastatic renal-cell carcinoma. Groupe Français d'Immunothérapie.. N Engl J Med.

[PDF_00724] Patel N. P., Lavengood R. W. (1978). Renal cell carcinoma: natural history and results of treatment.. J Urol.

[PDF_00726] Reiter Z., Ozes O. N., Blatt L. M., Taylor M. W. (1992). A dual anti-tumor effect of a combination of interferon-alpha or interleukin-2 and 5-fluorouracil on natural killer (NK) cell-mediated cytotoxicity.. Clin Immunol Immunopathol.

[PDF_00730] Rosenberg S. A., Lotze M. T., Muul L. M., Chang A. E., Avis F. P., Leitman S., Linehan W. M., Robertson C. N., Lee R. E., Rubin J. T. (1987). A progress report on the treatment of 157 patients with advanced cancer using lymphokine-activated killer cells and interleukin-2 or high-dose interleukin-2 alone.. N Engl J Med.

[PDF_00735] Rosenstein M., Ettinghausen S. E., Rosenberg S. A. (1986). Extravasation of intravascular fluid mediated by the systemic administration of recombinant interleukin 2.. J Immunol.

[PDF_00738] Schomburg A., Kirchner H., Fenner M., Menzel T., Poliwoda H., Atzpodien J. (1993). Lack of therapeutic efficacy of tamoxifen in advanced renal cell carcinoma.. Eur J Cancer.

[PDF_00741] Sella A., Kilbourn R. G., Gray I., Finn L., Zukiwski A. A., Ellerhorst J., Amato R. J., Logothetis C. J. (1994). Phase I study of interleukin-2 combined with interferon-alpha and 5-fluorouracil in patients with metastatic renal cell cancer.. Cancer Biother.

[PDF_00745] Sleijfer D. T., Janssen R. A., Buter J., de Vries E. G., Willemse P. H., Mulder N. H. (1992). Phase II study of subcutaneous interleukin-2 in unselected patients with advanced renal cell cancer on an outpatient basis.. J Clin Oncol.

[PDF_00749] Stahl M., Wilke H. J., Seeber S., Schmoll H. J. (1992). Cytokines and cytotoxic agents in renal cell carcinoma: a review.. Semin Oncol.

[PDF_00751] Tourani J. M., Pfister C., Berdah J. F., Benhammouda A., Salze P., Monnier A., Paule B., Guillet P., Chretien Y., Brewer Y. (1998). Outpatient treatment with subcutaneous interleukin-2 and interferon alfa administration in combination with fluorouracil in patients with metastatic renal cell carcinoma: results of a sequential nonrandomized phase II study. Subcutaneous Administration Propeukin Program Cooperative Group.. J Clin Oncol.

[PDF_00765] Wadler S., Schwartz E. L. (1990). Antineoplastic activity of the combination of interferon and cytotoxic agents against experimental and human malignancies: a review.. Cancer Res.

[PDF_00768] West W. H., Tauer K. W., Yannelli J. R., Marshall G. D., Orr D. W., Thurman G. B., Oldham R. K. (1987). Constant-infusion recombinant interleukin-2 in adoptive immunotherapy of advanced cancer.. N Engl J Med.

[PDF_00771] Wirth M. (1991). The current use of interferons, interleukin-2 and tumor necrosis factor in renal cell cancer.. Urol Int.

[PDF_00760] van Herpen C. M., Jansen R. L., Kruit W. H., Hoekman K., Groenewegen G., Osanto S., De Mulder P. H. (2000). Immunochemotherapy with interleukin-2, interferon-alpha and 5-fluorouracil for progressive metastatic renal cell carcinoma: a multicenter phase II study. Dutch Immunotherapy Working Party.. Br J Cancer.

